# Diagnostic Dilemma in an Infant With Sound‐Triggered Motor Events: Reflex Epilepsy Versus Exaggerated Startle—A Case Report

**DOI:** 10.1002/ccr3.72594

**Published:** 2026-04-20

**Authors:** Aakash Pandit, Melisha Koirala, Anil Shahi, Anupama Sharma, Aashish Panta, Shuvra Rimal

**Affiliations:** ^1^ Department of Internal Medicine Chitwan Medical College Bharatpur Nepal; ^2^ Department of Internal Medicine National Medical College Birgunj Nepal

**Keywords:** auditory‐triggered seizures, electroencephalography, epileptic encephalopathy, exaggerated startle reflex, infantile epilepsy, pediatrics and adolescent medicine reflex epilepsy, sleep‐activated epileptiform discharges

## Abstract

Auditory‐triggered motor events in infancy present a significant diagnostic challenge due to overlap between epileptic and non‐epileptic startle phenomena. We report the case of a term female infant with neonatal‐onset seizures and subsequent development of reproducible sound‐triggered jerky movements, raising diagnostic uncertainty between exaggerated startle reflex and reflex auditory epilepsy. The child initially presented with seizures on the third day of life and was treated with phenobarbital. During early infancy, brief motor events consistently precipitated by sudden auditory stimuli were observed, later accompanied by spontaneous seizures and developmental plateauing. Serial electroencephalography (EEG) revealed evolution from focal frontotemporal interictal epileptiform discharges to multifocal epileptiform activity with marked sleep activation, while repeated brain magnetic resonance imaging remained structurally normal. Over time, global developmental delay with hypotonia and impaired motor milestones became evident. The electroclinical trajectory suggested an evolving epileptic encephalopathy within the epileptic encephalopathy with spike–wave activation in sleep spectrum, despite the absence of a classic continuous spike‐and‐wave during sleep pattern. Whole‐exome sequencing identified a homozygous HEXA gene variant of uncertain significance, without definitive biochemical or clinical evidence of GM2 gangliosidosis. The child was managed with multiple antiseizure medications and supportive neurodevelopmental interventions, resulting in partial seizure control but persistent developmental impairment. This case underscores the importance of longitudinal electroclinical correlation in infants with stimulus‐triggered motor events and highlights that reproducible auditory‐induced events with evolving epileptiform EEG abnormalities and developmental impact strongly favor reflex auditory epilepsy over non‐epileptic exaggerated startle, even in the setting of normal neuroimaging and genetic uncertainty.

## Key Clinical Message

Auditory‐triggered jerks in infancy require careful differentiation between non‐epileptic hyperekplexia and reflex epilepsy. While interictal EEG discharges and neurodevelopmental plateau suggest an evolving epileptic encephalopathy, definitive diagnosis ideally requires ictal video‐EEG to correlate motor events with electrographic discharges.

## 1 Introduction

Reflex epilepsies are a distinct subset of epileptic disorders in which seizures are consistently precipitated by specific external stimuli, including sensory inputs such as light, somatosensory stimulation, or unexpected sound. These provoked seizures are mediated by hyperexcitable cortical networks that transiently respond to afferent sensory input with synchronous epileptiform discharges. Reflex seizures may occur as isolated events or as part of broader epilepsy syndromes with spontaneous seizures. The classification of reflex seizures includes both generalized and focal reflex phenomena; triggers can include visual stimuli (e.g., flickering light), reading or cognitive tasks, somatosensory input, and acoustic or startle‐evoking auditory stimuli. Electroencephalography (EEG) findings in reflex epilepsies generally demonstrate epileptiform abnormalities in interictal or ictal recordings that correlate with the clinical seizure events. Reflex epilepsies are considered rare, representing a minority of all epileptic disorders, but are important due to their reproducibility and implications for management and avoidance strategies [1].

Among reflex epilepsies, seizures triggered by acoustic or startle stimuli, often referred to as startle epilepsy or auditory reflex epilepsy, have been described across a range of ages from infancy through adulthood. In startle epilepsy, sudden unexpected noise or tactile input elicits brief seizures, which may be generalized or focal in onset. These events are clinically characterized by rapid onset of myoclonic, tonic, or tonic–clonic activity that follows the startling stimulus, and by reproducibility with trigger exposure. Interictal EEG abnormalities, including focal or multifocal epileptiform discharges, are frequently observed in these patients, and ictal EEG may demonstrate diffuse changes or electrodecremental patterns during the event. Structural brain abnormalities, perinatal insults, and other underlying pathologies are common in larger case series, although reflex seizures can also occur in idiopathic or generalized epilepsy syndromes [2, 3].

In infancy, brief motor jerks or startle‐like movements triggered by noise can be particularly challenging to classify. These may represent epileptic reflex events, but they can also overlap phenomenologically with non‐epileptic startle disorders such as hyperekplexia—a rare neurogenetic condition characterized by an exaggerated startle reflex in response to auditory, tactile or other sensory stimuli. Hyperekplexia presents in the neonatal or early infant period with excessive startle responses and increased muscle tone, and can be misdiagnosed as epileptic seizures. Unlike epileptic events, hyperekplexia typically does not have an electrographic correlate on EEG during startle episodes. Genetic mutations affecting glycinergic inhibitory neurotransmission (e.g., in GLRA1, SLC6A5, GLRB, GPHN) have been identified in many cases. Early recognition of hyperekplexia is important because appropriate therapy (e.g., clonazepam) differs markedly from antiseizure medications used for epilepsy [4, 5].

Distinguishing reflex auditory epileptic seizures from exaggerated startle responses/non‐epileptic startle behavior relies on careful clinical observation, documentation of reproducibility and phenomenology, and—critically—EEG correlation during events.

Here we present the case of a female infant with early‐onset, stimulus‐dependent motor events consistently triggered by auditory stimuli, initially raising diagnostic uncertainty between exaggerated startle reflex and reflex epilepsy. The clinical course was notable for neonatal seizures, evolution of sound‐triggered jerks, abnormal serial electroencephalographic findings with sleep‐activated epileptiform discharges, and emerging neurodevelopmental delay despite normal neuroimaging. This case highlights the challenges in distinguishing reflex auditory epilepsy from non‐epileptic startle disorders in infancy and underscores the importance of longitudinal electroclinical correlation in recognizing evolving epileptic encephalopathy.

## 2 Case History/Examination

The patient is a female infant, born at term via lower‐segment cesarean section with a birth weight of 2.9 kg. She cried immediately at birth, and the perinatal period was uneventful. Maternal history was notable for TORCH seropositivity, for which spiramycin was administered during pregnancy.

On day 3 of life, the neonate experienced stiffening followed by rhythmic jerking and was started on phenobarbital (18 mg PO once daily; 4 mg/kg/day). Initial evaluation, including cranial ultrasonography, ophthalmologic examination, and routine laboratory tests, was unremarkable. Neonatal TORCH serology showed positive IgG and IgM antibodies for rubella, CMV, and HSV‐1/2. Quantitative urinary CMV DNA PCR was negative. Liver and renal function tests were within normal limits, and thyroid function was normal.

At approximately 45 days of life, caregivers observed brief, sudden symmetric tonic extension of the limbs precipitated by loud auditory stimuli. These episodes were short, stereotyped, and reproducible. There was no loss of consciousness or postictal lethargy. Between 4 and 6 months, developmental progress plateaued, with features of hypotonia, poor trunk control, and delayed motor milestones. Physical examination revealed normal systemic findings, normal muscle bulk, and absence of dysmorphic features or neurocutaneous markers.

Subsequent neurological examinations confirmed trunk‐predominant hypotonia and delayed gross motor development, including partial head control and inability to roll independently at 6 months. Reflexes were preserved, and deep tendon reflexes were normal. Ophthalmoscopy revealed cherry‐red macula.

## 3 Methods (Investigations and Treatment)

### 3.1 Electroencephalography (EEG)

At 6 months 15 days: induced sleep EEG showed frequent left frontotemporal interictal epileptiform discharges with secondary generalization; arousal was reactive, no ictal event recorded. At 15 months, EEG showed abundant multifocal interictal epileptiform discharges over posterior regions with sleep activation and attenuation bursts lasting 4–5 s. No ictal events were observed.

### 3.2 Neuroimaging

Cranial ultrasonography at 1 month: normal cerebral hemispheres, ventricles, basal ganglia, posterior fossa structures, and no evidence of hemorrhage. MRI brain at 6 months and 15 months: normal morphology, signal intensity, and myelination appropriate for age. No focal lesions, cortical malformations, or structural abnormalities were detected.

### 3.3 Cardiac and Abdominal Evaluation

Echocardiography: ostium secundum ASD 2.5 mm, left‐to‐right shunt, normal chamber size and pulmonary artery pressure. Abdominal ultrasonography: normal liver size and echotexture; no splenomegaly at initial assessment.

### 3.4 Laboratory Investigations

Routine hematological and liver function tests, renal investigations were within normal limits for age. Thyroid function was normal. TORCH and genetic testing: neonatal serology positive for rubella, CMV, HSV‐1/2. Whole‐exome sequencing detected a homozygous HEXA missense variant (c.674G>A; p.Gly225Glu) classified as a variant of uncertain significance.

### 3.5 Treatment

Neonatal seizures: phenobarbital. Later seizures: levetiracetam (30 mg/kg/day, adjusted by age), oxcarbazepine (initially 13 mg/kg/day, then 37.5–75 mg/day), short course of phenytoin (tapered). Physiotherapy: trunk control, rolling facilitation, core strengthening, play‐based sensory stimulation. Gastroenterology consult for constipation; caregiver education on trigger avoidance for auditory stimuli.

## 4 Conclusion and Results (Outcome and Follow‐Up)

The patient continued to experience auditory‐triggered seizures despite optimized therapy, with a reduction in spontaneous seizure frequency following medication adjustments. Developmental delay persisted, predominantly in gross motor and language domains, necessitating ongoing neurodevelopmental support.

Serial EEGs demonstrated progression from focal frontotemporal interictal discharges to multifocal posterior spike–wave discharges with sleep activation, consistent with evolving epileptic encephalopathy. Neuroimaging remained normal.

Follow‐up included continued antiseizure therapy, physiotherapy, and multidisciplinary management. Genetic counseling was recommended for the HEXA variant, with suggestions for enzymatic confirmation and family segregation studies. The findings indicate reflex auditory seizures with subsequent spontaneous seizures and developmental impact, suggesting a clinical presentation consistent with reflex epilepsy evolving into an epileptic encephalopathy, rather than a primary exaggerated startle response.

## 5 Discussion

This case highlights the diagnostic complexity of sound‐triggered paroxysmal motor events in infancy and the importance of integrating clinical observation with EEG and pattern recognition to differentiate epileptic versus non‐epileptic phenomena.

In our patient, the reproducible auditory precipitant (loud sounds) associated with brief jerks raised early suspicion for a reflex phenomenon. However, the presence of interictal epileptiform discharges on EEG including initially focal left frontotemporal discharges and later multifocal discharges with sleep activation is highly suggestive of a cortical epileptic mechanism, rather than a benign non‐epileptic startle. Reflex seizures often demonstrate abnormal EEG interictally or ictally, distinguishing them from non‐epileptic paroxysms [1].

Consequently, a primary exaggerated startle disorder such as hyperekplexia was clinically differentiated from our patient's presentation. Unlike reflex epilepsies, hyperekplexia is a non‐epileptic, brainstem‐mediated condition classically characterized by sustained generalized muscle stiffness, resistance to habituation, and a strictly normal EEG during episodes [4, 5, 6, 7]. Our patient's presentation of brief, non‐sustained jerks, combined with the progressive focal‐to‐multifocal epileptiform EEG abnormalities, appeared less consistent with a primary neurotransmission defect and more aligned with the trajectory of an evolving epileptic encephalopathy.

Several clinical reports describe reflex myoclonic epilepsy of infancy and reflex seizures induced predominantly by auditory or tactile stimuli, often with normal neuroimaging and variable EEG features; some infants may exhibit generalized spike–wave discharges during events, supporting an epileptic mechanism [8, 9].

In this child, the evolution of EEG findings from focal discharges to multifocal patterns with sleep activation combined with neurodevelopmental delay suggests an underlying epileptic encephalopathy, in which epileptiform activity contributes to developmental slowing. Sleep‐activated epileptiform discharges are recognized in epileptic encephalopathies such as CSWS/ESES and are associated with cognitive and motor impairment beyond seizure frequency per se. Although the classic CSWS EEG pattern (continuous spike–wave during sleep) was not documented formally, the sleep‐activated multifocal discharges align with electrophysiological patterns linked to developmental impacts in pediatric epilepsy [10].

A review of similar pediatric cases reveals that auditory‐triggered reflex seizures are frequently associated with underlying structural or metabolic pathologies, though idiopathic cases exist [2, 9]. While many reported cases of reflex myoclonic epilepsy of infancy describe a benign course with age‐dependent resolution, our patient's progression toward multifocal discharges and developmental plateau aligns more closely with ‘symptomatic’ startle epilepsy, which often carries a guarded neurodevelopmental prognosis [8, 9, 11].

The identification of a HEXA homozygous variant of uncertain significance necessitates cautious interpretation. While HEXA mutations cause GM2 gangliosidosis (Tay‐Sachs disease), a neurodegenerative condition that can include abnormal startle responses, clinical and imaging features typical of classical Tay–Sachs were not substantiated in this case. Symptomatic hyperekplexia or exaggerated startle sign has been reported in metabolic and neurodegenerative disorders, but such phenomena in these conditions are not directly equivalent to reflex epileptic seizures and usually follow a different progression and associated findings [11].

Finally, neonatal seizures in the context of maternal TORCH seropositivity prompted initial evaluation for congenital infection. However, negative CMV PCR, normal neuroimaging, and absence of consistent clinical signs of active infection argue against congenital viral encephalitis as the primary cause of the evolving epileptic phenotype.

In summary, the combination of reproducible auditory triggers, abnormal EEG evolution, and developmental delay points toward a likely diagnosis of reflex auditory epilepsy with evolving epileptic encephalopathy rather than a primary non‐epileptic startle disorder. EEG remains indispensable in differentiating epileptic from non‐epileptic paroxysms and guiding management in infants with stimulus‐associated motor events.

Structured Differential Diagnosis of Auditory‐Triggered Motor Paroxysms in Infancy.
Reflex Auditory (Startle) Epilepsy.
Pathophysiology: Cortical hyperexcitability responding to afferent sensory input [1].
Motor Phenomenology: Brief, rapid onset myoclonic, tonic, or tonic–clonic jerks. Lacks sustained stiffness.
EEG Findings: Interictal focal/multifocal epileptiform discharges. Ictal recordings may show discharges or electrodecremental patterns.
Relevance to Current Case: Matches our patient's brief jerks, evolving focal‐to‐multifocal EEG with sleep activation, and developmental plateau [10].
Primary Hyperekplexia.
Pathophysiology: Brainstem‐mediated glycinergic inhibitory neurotransmission defect [4].
Motor Phenomenology: Exaggerated startle followed by transient, sustained tonic stiffness.
EEG Findings: Strictly normal during startle episodes; lacks epileptiform correlates [5].
Relevance to Current Case: Ruled out in our patient due to the presence of progressive epileptiform EEG abnormalities and lack of sustained stiffness.
GM2 Gangliosidosis (e.g., Tay‐Sachs Disease).
Pathophysiology: Lysosomal storage disorder with accumulation of GM2 gangliosides [11].
Motor Phenomenology: Symptomatic exaggerated startle alongside progressive motor decline.
EEG Findings: Variable; can show progressive slowing or paroxysmal activity later in the disease course.
Relevance to Current Case: Our patient possesses a HEXA VUS and a cherry‐red macula, necessitating careful longitudinal follow‐up despite atypical progression.

A limitation of this case is the absence of ictal video‐EEG documentation to definitively confirm the epileptic nature of the startle‐induced events. However, the interictal progression and developmental trajectory strongly support the proposed diagnosis.

## Author Contributions

**Aakash Pandit:** conceptualization, data curation, formal analysis, investigation, methodology, project administration. **Melisha Koirala:** conceptualization, data curation, formal analysis. **Anil Shahi:** conceptualization, data curation, formal analysis. **Anupama Sharma:** conceptualization, data curation, formal analysis, investigation. **Aashish Panta:** conceptualization, data curation, formal analysis, investigation. **Shuvra Rimal:** conceptualization, data curation, formal analysis.

## Funding

The authors have nothing to report.

## Consent

Written informed consent was obtained from the patient's parents/legal guardian for publication and any accompanying images. A copy of the written consent is available for review by the Editor‐in‐Chief of this journal on request.

## Conflicts of Interest

The authors declare no conflicts of interest.

## Data Availability

The data that support the findings of this study are available from the corresponding author upon reasonable request.

## References

[1] L. Y. Xue and A. L. Ritaccio , “Reflex Seizures and Reflex Epilepsy,” American Journal of Electroneurodiagnostic Technology 46, no. 1 (2006): 39–48. 16605171

[2] Z. Yang , X. Liu , J. Qin , et al., “Clinical and Electrophysiological Characteristics of Startle Epilepsy in Childhood,” Clinical Neurophysiology 121, no. 5 (2010): 658–664, 10.1016/j.clinph.2009.12.020. 20071222

[3] G. Rubboli , B. Kassabian , and E. Gardella , “Reflex Epilepsies With Auditory and Somatosensory Precipitants,” in Reflex Epilepsies for the Practicing Clinician, ed. C. N. Joshi and R. Guerrini (Oxford Academic, 2025), 10.1093/med/9780197634547.003.0003.

[4] R. Falsaperla , V. Sortino , V. Giacchi , et al., “Neonatal Hyperekplexia: Is It Still a Diagnostic Challenge? Evidence From a Systematic Review,” Journal of Child Neurology 39, no. 11–12 (2024): 415–424, 10.1177/08830738241273425. 39223854

[5] S. C. Dudipala , R. V. Reddy , and R. Shankar , “Hyperekplexia: A Treatable Seizure Mimicker in Infants,” Cureus 15, no. 4 (2023): e38082, 10.7759/cureus.38082. 37252475 PMC10208903

[6] J. Gupta , S. Badal , V. Anand , P. Jauhari , B. Chakrabarty , and S. Gulati , “Hyperekplexia: A Frequent Near Miss in Infants and Young Children,” Neurology India 70, no. 1 (2022): 312–314, 10.4103/0028-3886.338670. 35263902

[7] C. P. Panayiotopoulos , “Chapter 5, Neonatal Seizures and Neonatal Syndromes,” in The Epilepsies: Seizures, Syndromes and Management (Bladon Medical Publishing, 2005), https://www.ncbi.nlm.nih.gov/books/NBK2599/. 20821848

[8] D. Zafeiriou , E. Vargiami , and E. Kontopoulos , “Reflex Myoclonic Epilepsy in Infancy: A Benign Age‐Dependent Idiopathic Startle Epilepsy,” Epileptic Disorders 5, no. 2 (2003): 121–122. 12875957

[9] S. Ricci , R. Cusmai , L. Fusco , and F. Vigevano , “Reflex Myoclonic Epilepsy in Infancy: A New Age‐Dependent Idiopathic Epileptic Syndrome Related to Startle Reaction,” Epilepsia 36, no. 4 (1995): 342–348, 10.1111/j.1528-1157.1995.tb01007.x. 7607111

[10] I. Sánchez Fernández , K. E. Chapman , J. M. Peters , C. Harini , A. Rotenberg , and T. Loddenkemper , “Continuous Spikes and Waves During Sleep: Electroclinical Presentation and Suggestions for Management,” Epilepsy Research and Treatment 2013 (2013): 583531, 10.1155/2013/583531. 23991336 PMC3748771

[11] P. Banyal , M. Arora , and A. G. Saini , “Symptomatic Hyperekplexia: An Important Clue to Neurodegeneration in Children,” BMJ Case Reports 16 (2023): e256628. 10.1136/bcr-2023-256628 PMC10618968 37899085

